# Psychological distress and well-being among students of health disciplines in Geneva, Switzerland: The importance of academic satisfaction in the context of academic year-end and COVID-19 stress on their learning experience

**DOI:** 10.1371/journal.pone.0266612

**Published:** 2022-04-06

**Authors:** Nguyen Toan Tran, Jessica Franzen, Françoise Jermann, Serge Rudaz, Guido Bondolfi, Paolo Ghisletta

**Affiliations:** 1 Faculty of Medicine, University of Geneva, Geneva, Switzerland; 2 Australian Centre for Public and Population Health Research, Faculty of Health, University of Technology Sydney, Sydney, NSW, Australia; 3 School of Health Sciences, Geneva HES-SO, University of Applied Sciences and Arts Western Switzerland, Geneva, Switzerland; 4 Department of Psychiatry, Geneva University Hospitals, Geneva, Switzerland; 5 School of Pharmaceutical Sciences, University of Geneva, Geneva, Switzerland; 6 Faculty of Psychology, University of Geneva, Geneva, Switzerland; 7 Faculty of Psychology, UniDistance Suisse, Brig, Switzerland; 8 Swiss National Centre of Competence in Research LIVES, University of Geneva, Geneva, Switzerland; Public Library of Science, UNITED STATES

## Abstract

**Introduction:**

University students’ psychological health is linked to their academic satisfaction. This study aimed to investigate students’ psychological health and academic satisfaction in the context of COVID-19 and academic year-end stress.

**Materials and methods:**

Standardized self-filled scales for anxiety, depression, stress, psychological well-being, academic satisfaction (subjective assessment of students’ quality of life in their educational setting), and an ad-hoc scale for stress on the learning experience due to COVID-19 were used in this cross-sectional study. Participants were first- to third-year students of eight different health-related tracks in Geneva, Switzerland. Descriptive statistics and hierarchical regression analyses were applied.

**Results:**

In June 2020, out of 2835 invited students, 433 (15%) completed the survey. Academic satisfaction was a stronger mental health predictor than COVID-19 stress on the learning experience, which mainly predicted stress and anxiety. Lower academic satisfaction scores were significantly associated with stress (β = −0.53, p < 0.001), depression (β = −0.26, p < 0.001), anxiety (β = −0.20, p < 0.001), while higher scores with psychological well-being (β = 0.48, p < 0.001). Identifying as female was strongly associated with anxiety and stress but not with depression or psychological well-being. Lower age was associated with stress only. The nature of the academic training had a lesser impact on mental health and the academic year had no impact.

**Conclusions:**

Academic satisfaction plays a more substantial role than COVID-19 stress on the learning experience in predicting students’ overall mental health status. Training institutions should address the underlying factors that can enhance students’ academic satisfaction, especially during the COVID-19 period, in addition to ensuring that they have a continuous and adequate learning experience, as well as access to psychosocial services that help them cope with mental distress and enhance their psychological well-being.

## Introduction

Psychological well-being comprises the dimensions of self-acceptance, positive relations with others, autonomy, environmental mastery, purpose in life, and personal growth [1]. Psychological distress is usually defined as a state of emotional suffering consisting of symptoms related to depression and anxiety [2]. Both psychological well-being and distress should be considered when researching student mental health, not least because such a holistic approach aligns with the World Health Organization’s definition of mental health: *a state of well-being in which an individual realizes his or her own abilities*, *can cope with the normal stresses of life*, *can work productively and is able to make a contribution to his or her community* [3].

Most of the research in health sciences training has centered on the psychological distress of medical and nursing students: a systematic review of the literature found among medical students a very high depression and anxiety prevalence and a higher psychological distress level than in the general population [4]. Several studies showed that nursing students report very high anxiety, stress, and depression scores, and more stress, anxiety, and depression than students from other disciplines and people in the labor force [5–7]. Among nursing students, clinical practice, theoretical training, personal life, and social life were identified as four causes of stress in a qualitative study [8], whereas clinical practice was established as the primary stressor in other research [5, 9–11].

There is limited literature on student mental health, specifically at the end of the academic year. In contrast, exams and tests, which often intensify during the academic year-end, represent a well-established source of stress among students [12–14]. For some students, the primary cause of stress is examinations and the subsequent wait for results, often at the end of the academic year [15]. Undergraduate medical students reported that exams and academic concerns were among the most common and severe stress sources [16]. Common factors of exam anxiety include extensive course loads and lack of physical activities [17]. Pre-examination stress is also widespread and can manifest, for example, in changed concentration span, disturbed sleep, irritability, mood swings, anorexia, or fatigue, as evidenced by a study among second-year medical students [13].

With regard to research on pyschological well-being, a study showed that the majority of students had a good quality of life and were satisfied with their health and way of life [5]. Another study found a relationship between nursing students’ psychological well-being and physical activity [18]. The majority of the body of research investigating contributing factors has examined risk factors for increased psychological distress. An important factor for anxiety, depression, and stress is female identity. Overall, students identifying as female show higher levels of anxiety and stress than male students [19]. In terms of general psychological distress, the same is true for students identifying as female in health-related disciplines [4, 7]. Another study did not confirm this gender gap [6]. The academic year is also a decisive factor: first-year and second-year students are more stressed, depressed, and anxious than others (due, among other things, to higher student dropout rates earlier in the curriculum) [8, 10, 19–22], and fourth-year students have lower depression scores than second and third-year students [5]. Only a few studies examined protective factors in comparison to the numerous ones on risk factors. Internal and external factors predicting psychological well-being in nursing students were investigated in one study [23, 24]. Self-efficacy, resilience, mindfulness skills, and social support were found to have a positive impact on their psychological well-being. Recently, our research team highlighted in Franzen et al. (2021) the importance of academic satisfaction (defined as a subjective assessment of students’ quality of life in their educational setting) as the most powerful predictor of depression, anxiety, stress, and psychological well-being among students of health disciplines.

In March 2020, the COVID-19 pandemic locked down Western Europe. The pandemic has affected the mental health of the general population across the globe, as illustrated by a systematic review and meta-analysis of studies published in the early months of the pandemic (until May 2020) and conducted in ten countries across Asia, Europe, and Africa. This analysis showed a pooled prevalence of stress equal to 30%, of anxiety to 32%, and of depression to 34% [25].

COVID-19 abruptly closed schools and universities, upending students’ in-person learning and living conditions. Reflecting the widespread effects of health-related fears, uncertainty, and downstream academic consequences, studies have reported negative impacts on students’ mental health. For example, a large cohort study involving initially 164,101 college students in China showed a prevalence of stress of 35% during the acute phase of the outbreak, decreasing to 16% two months later as the outbreak subsided [26]. However, during the same period, depression increased from 22% to 26% and anxiety from 11% to 15%. Less physical exercise, lower social support, and a dysfunctional family negatively worsened students’ psychological distress along with COVID-19 related worries and knowledge of confirmed or suspected cases in their community. A mixed-method study among 195 US college students carried out in April 2020, a month after the stay-at-home order, indicated that 71% of students reported increased stress and anxiety related to the outbreak [27].

As for health discipline students, a nationwide survey conducted in April 2020 in Saudi Arabia among dentistry students reported a high prevalence of depression (61%), followed by anxiety (37%) and stress (35%), all of which affected more students identifying as female living alone and junior students than others [28]. In Israel, a study during the third week of the national lockdown among nursing students showed that the prevalence of moderate and severe anxiety was 43% and 13%, respectively, compounded by female identity and lack of personal protective equipment [29].

In Switzerland, the COVID-19 Stringency Index, a composite indicator including school closures, workplace closures, gathering restrictions, and travel bans was still at 0 (no measure) out of 100 (strictest measures) until February 24, 2020, when it sharply climbed to 73 on March 17. April 28 marked the beginning of the Index decline, reaching 35 on June 30, 2020 [30]. In line with the new measures announced by the Swiss authorities to stem COVID-19 transmission, the University of Geneva, where our study takes place, suspended all in-person teaching from March 2020 onward. All teaching moved online for most of the remaining semester.

In response to the COVID-19 pandemic and with the aim to investigate whether the pandemic could be associated with students’ mental health deterioration, our group launched in June 2020, which also corresponded to the academic year-end, a similar survey to the one we did among students of health disciplines in October 2019 (pre-pandemic, beginning of the academic year) (Franzen et al., 2021) [24]. This second survey, which was not planned when we conceived the first survey, aimed to investigate students’ psychological health and academic satisfaction in the context of COVID-19 and academic year-end stress. Therefore, our first study and second (a posteriori) study are independent and have unpaired data. Nonetheless, comparison between the two is done where relevant in the Results and Discussion sections.

## Materials and methods

### Study population, setting, and sampling

Bachelor’s degree students of the 2019–2020 academic year from the University of Geneva’s Psychology Department, the Faculty of Medicine, the School of Pharmaceutical Sciences, and the School of Health Sciences Geneva (an applied university with courses in midwifery, nursing, physiotherapy, nutrition and dietetics, and medical radiology technology) were enrolled in this cross-sectional study. During the month of June 2020, close to the academic year-end, all students were invited to participate in the study, which used random sampling stratified by health disciplines. The enrollment had no exclusion criteria. On the basis of the 2835 Bachelor’s degree students registered for the academic year and considering a 95% confidence interval, 0.5 standard deviation, and 5% margin of error, our calculated ideal sample size had to include at least 339 participants.

### Measurements

The study collected focused sociodemographic data (gender identity, age, health discipline, and current academic year), and applied the following scales for perceived stress, anxiety, depression, psychological well-being, satisfaction with studies, and stress due to COVID-19.

#### Depression and anxiety

To detect the presence of depression and anxiety symptoms and measure their severity, we used the Hospital Anxiety and Depression Scale (HADS), which comprises a depression subscale and an anxiety subscale, each with seven items rated from 0 to 3 [31, 32]. Scores can range from 0 to 21, with higher scores reflecting greater levels of anxiety or depression. A score of 8 or above indicates the presence of depression or anxiety [31]. The HADS was previously used among medical students in a longitudinal study over six years [33]. The HADS Cronbach’s alpha for our study sample was 0.84 for the depression subscale and .84 for the anxiety subscale.

#### Perceived stress

To measure perceived stress or the degree to which they typically view life circumstances as threatening, participants rated statements of the 14-item Perceived Stress Scale (PSS) by Cohen et al. on a 0 (never) to 4 (very often) scale [34]. The PSS has been widely used, including in studies involving psychology or medical students [35]. The PSS Cronbach’s alpha for our study sample was 0.88.

#### Psychological well-being

To measure psychological well-being, participants rated statements of the Psychological Well-Being (PWB) Scale from 1 (disagree) to 6 (agree) [1, 36]. Autonomy, environmental mastery, personal growth, positive relationships with others, purpose in life, and self-acceptance are the six dimensions of this 18-item scale. The PWB was used in studies involving psychology students, among others [37]. The PWB Cronbach’s alpha for our study sample was 0.78.

#### Academic satisfaction

To measure student’s academic satisfaction, participants rated statements of the Scale of Satisfaction with Studies (SSS) from 1 (strongly disagree) to 7 (strongly agree) [38]. This scale comprises five items (such as “I am satisfied with my academic life” or “In general, my academic life closely matches my ideals”) that capture the overall and subjective assessment of students’ quality of life in their learning environment. We could not identify studies using this scale with students from health disciplines. The SSS Cronbach’s alpha for our study sample was 0.88.

#### Additional stress on learning experience due to COVID-19

Using a visual analog scale, participants answered the following questions: “To what extent has the COVID-19 situation put additional stress on your learning experience?” Answers ranged from 0 (no additional stress) to 10 (severe additional stress).

### Data collection

In June 2020, close to the academic year-end, the various school secretariats sent e-mails to all enlisted students to invite them to take part in the study. Those interested logged onto a secure electronic site (EvaSys Education Survey Automation Suite version 7.1, Electric Paper Evaluation Systems GmbH, Lüneburg, Germany) to give informed consent before completing the survey. The main investigator managed data in a confidential and safe manner through EvaSys and archived all data on a hard drive located in a secured office.

### Statistical analysis

Descriptive statistics were computed for demographic data as means and standard deviations (SD). Multiple hierarchical linear regression analyses were performed to quantify the contribution of these possible determinants on scales for depression, anxiety, stress, psychological well-being, and COVID-19 stress on learning experience. Five separate blocks of independent variables were established. The sequential entry of predictors was derived from past studies and comprised gender identity and age (block 1) [4, 7, 19], the academic years of training (block 2) [5, 8, 10, 19], the health disciplines (block 3) [5], academic satisfaction (block 4) [19], and finally COVID-19 stress on learning experience (block 5) [25–29]. Significance was defined by a p-value ≤ 0.05. *R*^*2*^ increase was examined to assess the increase in effect size between two successive blocks. To compare results between October 2019 and June 2020 studies, the chi-square test was used for gender identity and t-test for age and the mental health scales with statistical significance determined by p ≤ 0.05. No data were missing as the electronic survey would not proceed if students did not answer each of the questions. Students had the option of interrupting their participation at any time during the survey. SPSS, version 25 (IBM, Armonk, NY, USA), was used for all the analyses.

### Ethics statement

The Geneva University Hospitals’ Ethics Research Committee reviewed the research protocol and decided to waive the need for an institutional review board as the study involved students and was anonymous (reference number: 2019–00696).

## Results

The survey was completed between June 2–29, 2020, by 433 (15%) out of 2835 students asked to participate in the research. There was no invalid or missing data. Gender identity, age, and scores of the depression/HADS, anxiety/HADS, stress/PSS, psychological well-being/PWB, academic satisfaction/SSS scales are presented in Table 1.

**Table 1:** Gender identity, age, health disciplines, and questionnaires scores (means (sd)), and comparison between June 2020 and October 2019 (Franzen et al., 2021).

Variables	June 2020 (n = 433)	October 2019 (n = 915) (Franzen et al., 2021)	Cohen’s d	Chi-square/t-test value	p-value
Identifying as female	357 (82.4%)	753 (82.3%)	--	0.01	0.95
Age	22.91 (4.05)	22.15 (4.25)	0.18	-3.09	0.002
Mental health scales					
Depression (HADS)	5.75 (4.38)	5.04 (3.62)	0.18	-3.14	0.002
Anxiety (HADS)	10.21 (4.80)	9.19 (4.45)	0.22	-3.82	<0.001
Stress (PSS)	28.06 (9.60)	25.59 (9.00)	0.27	-4.60	<0.001
Psychological well-being (PWB)	82.95 (11.03)	82.75 (11.14)	0.02	-0.32	0.75
Academic satisfaction (SSS)	21.90 (7.79)	23.24 (6.91)	-0.19	3.19	0.001
Additional stress due to COVID-19	4.91 (2.70)	Not applicable			

Notes: PWB: Psychological Well-Being Scale; SSS: Scale of Satisfaction with Studies; HADS: Hospital Anxiety and Depression Scale; PSS: Perceived Stress Scale.

Women formed the vast majority of respondents (n = 357, 82%). This reflected the majority identifying as female of the overall sampling pool—for example, the proportion of students identifying as female was 64% in the Faculty of Medicine and 81% in the Psychology Department. Participants’ age ranged from 16 to 62 years, with a mean age of 23, (Faculty of Medicine and School of Pharmaceutical Sciences) to 24 years (School of Health Sciences Geneva).

Participants were not only psychology, pharmaceutical sciences, and medical students, but also students of midwifery, nursing, physiotherapy, nutrition and dietetics, and medical radiology technology. We grouped together all the participants from the School of Health Sciences because a detailed analysis by discipline would have resulted in inadequate subsample sizes.

The linear hierarchical regressions results are summarized in Table 2. Blocks 1 to 3 predicted minimal amounts of variance for all outcomes (from less than 1% to 6%, mean amount = 3%). Academic satisfaction in block 4 was by far the strongest predictor, with *R*^*2*^ increases ranging from 14% to 29%. The addition of COVID-19 in block 5 contributed to a lesser extent, with higher *R*^*2*^ increases for stress (15%) and anxiety (13%) than depression (6%) and psychological well-being (3%). Lower academic satisfaction/SSS scores were strongly associated with more stress (β = −0.53, p < 0.001), depression (β = −0.26, p < 0.001), and anxiety (β = −0.20, p < 0.001), while higher satisfaction predicted greater psychological well-being (β = 0.48, p < 0.001).

**Table 2:** Hierarchical regression.

		Depression (HADS)		Anxiety (HADS)		Stress (PSS)		Psychological well-being (PWB)	
		B	SE	B	SE	B	SE	B	SE
Intercept		5.89***	0.23	10.71***	0.25	43.05***	0.50	82.49***	0.58
Block 1	Age	0.01	0.05	-0.002	0.06	-0.14	0.11	-0.09	0.13
	Gender identity	-0.76	0.55	-2.90***	0.60	-5.72***	1.18	2.60	1.39
	*R* ^*2*^	*0*.*004*		*0*.*05*		*0*.*06*		*0*.*009*	
Intercept		5.33***	0.39	10.08***	0.41	42.27***	0.83	82.66***	0.98
Block 1	Age	0.03	0.05	0.03	0.06	-0.08	0.12	-0.11	0.14
	Gender identity	-0.72	0.55	-2.87***	0.59	-5.71***	1.18	2.59	1.40
Block 2^¶^	1st year	1.25*	0.51	1.63**	0.54	2.42*	1.09	-0.52	1.29
	3rd year	0.34	0.54	0.16	0.57	-0.24	1.14	0.06	1.35
	Increase in *R*^*2*^	*0*.*02*		*0*.*02*		*0*.*02*		*0*.*001*	
Intercept		5.06***	0.49	9.61***	0.53	42.16***	1.06	83.46***	1.26
Block 1	Age	0.04	0.05	0.03	0.06	-0.10	0.12	-0.08	0.14
	Gender identity	-0.75	0.54	-2.84***	0.58	-5.58***	1.17	2.28	1.39
Block 2^¶^	1st year	1.42**	0.50	1.79**	0.54	2.83*	1.07	-0.93	1.28
	3rd year	0.26	0.52	0.14	0.56	-0.30	1.12	-0.07	1.34
Block 3^ǂ^	Medicine	-0.69	0.56	-0.59	0.61	-2.44*	1.22	2.16	1.45
	Pharmaceutical sciences	3.02***	0.69	2.79***	0.74	4.30**	1.49	3.83	1.78
	Psychology	0.13	0.53	0.68	0.56	0.18	1.13	-2.01	1.35
	Increase in *R*^*2*^	*0*.*06*		*0*.*05*		*0*.*04*		*0*.*03*	
Intercept		4.92***	0.41	9.49***	0.47	41.85***	0.87	83.71***	1.16
Block 1	Age	-0.02	0.05	-0.02	0.05	-0.23*	1.0	0.03	0.13
	Gender identity	-0.32	0.46	-2.46***	0.52	-4.60***	0.96	1.48	1.29
Block 2^¶^	1st year	0.66	0.43	1.10*	0.48	1.08	0.89	0.50	1.20
	3rd year	0.59	0.44	0.44	0.50	0.47	0.93	-0.69	1.24
Block 3^ǂ^	Medicine	0.30	0.48	0.31	0.55	-0.16	1.01	0.32	1.36
	Pharmaceutical sciences	1.48**	0.59	1.71*	0.67	1.57	1.24	-1.62	1.66
	Psychology	0.38	0.45	0.90	0.51	0.76	0.93	-2.48*	1.25
Block 4	Academic satisfaction	-0.30***	0.02	-0.28***	0.03	-0.70***	0.05	0.57***	0.07
	Increase in *R*^*2*^	*0*.*26*		*0*.*18*		*0*.*29*		*0*.*14*	
Intercept		2.27***	0.56	5.35***	0.60	32.87***	1.07	88.63***	1.62
Block 1	Age	-0.03	0.04	-0.04	0.05	-0.27**	0.08	0.05	0.13
	Gender identity	0.01	0.44	-1.94***	0.47	-3.42***	0.84	0.86	1.27
Block 2^¶^	1st year	0.30	0.41	0.54	0.44	-0.12	0.78	1.15	1.18
	3rd year	0.61	0.42	0.48	0.45	0.54	0.80	-0.73	1.22
Block 3^ǂ^	Medicine	0.42	0.46	0.51	0.50	0.26	0.88	0.08	1.33
	Pharmaceutical sciences	1.64**	0.57	1.41*	0.61	0.91	1.08	-1.26	1.63
	Psychology	0.28	0.43	0.75	56	42	0.81	-2.30	1.23
Block 4	Academic satisfaction	-0.26***	0.02	-0.20***	0.03	-0.53***	0.05	0.48***	0.07
Block 5	COVID-19 stress on learning experience	0.45***	0.07	0.70***	0.07	1.53***	0.13	-0.84***	0.20
	Increase in *R*^*2*^	*0*.*06*		*0*.*13*		*0*.*15*		*0*.*03*	

Note.

* p < 0.05

** p < 0.01

*** p < 0.001; ^¶^ reference: 2^nd^ year; ^ǂ^ reference: School of Health Sciences Geneva (including midwifery, nursing, physiotherapy, nutrition and dietetics, and medical radiology technology); gender identity was coded 0 = female, 1 = male; B: Beta coefficients; SE: standard error; HADS: Hospital Anxiety and Depression Scale; PSS: Perceived Stress Scale; PWB: Psychological Well-Being Scale; SSS: Scale of Satisfaction with Studies. Reference categories: women, second year, School of Health Sciences Geneva.

Higher scores on COVID-19 stress on learning experience were strongly associated with greater stress (β = 1.53, p < 0.001), anxiety (β = 0.70, p < 0.001), and depression (β = 0.45, p < 0.001), while less COVID-19 stress on learning experience predicted higher psychological well-being (β = −0.84, p < 0.001). Female identity was also strongly associated with higher stress (β = −3.42, p < 0.001) and anxiety (β = −1.94, p < 0.001), but not with depression or psychological well-being. Lower age was associated only with more stress (β = −0.27, p < 0.01). There were no marked differences between the different health disciplines in relation to stress and psychological well-being. However, pharmaceutical sciences students reported higher depression (β = 1.64, p < 0.01) and anxiety (β = 1.41, p < 0.005) compared to participants from other disciplines. The academic years did not predict any outcome.

When comparing the two independent groups that participated in the first survey in 2019 (pre-pandemic, academic year start) and the second survey in 2020 (per-pandemic, academic year-end), results showed that participants in 2020 were older by almost a year, although the difference was weak, as shown by its Cohen’s d value of 0.18 (Table 1). Although the differences between the two groups were statistically significant for depression, anxiety, stress, and academic satisfaction (p ≤ 0.002), these differences were weak as shown by their Cohen’s d values hovering around 0.2. There was no difference regarding psychological well-being.

## Discussion

This study sought to investigate the mental health status of Bachelor’s degree students of different health disciplines and related risk and protective factors in the context of COVID-19 and academic year-end. Year-end students reported lower academic satisfaction and were more stressed, anxious, and depressed than their counterparts at the beginning of the year. Year-end academic satisfaction had a critical impact on depression, anxiety, stress, and psychological well-being. COVID-19 stress on students’ learning experience was comparably a weaker predictor of their overall mental health; however, it still had some influence, but rather on stress and anxiety than depression and psychological well-being—the COVID-19 Stringency Index in Switzerland fell during the data acquisition timeframe from 55 (June 1) to 35 (June 30), a change that could have influenced these results, although classwork and exams continued online until the end of the academic year [30]. Women reported more anxiety and stress than men, decreased age was associated with stress, and the academic year had no influence. Pharmaceutical sciences students reported higher psychological distress more in the form of depression than stress.

The overall results converge with those of earlier studies in terms of gendered difference for anxiety and stress levels, however, with the caveat that our sample comprised an overwhelming proportion of participants identifying as female (82%). Convergence with the literature was also found in the positive relationship between students’ satisfaction and mental health [4, 7, 19, 39]. However, first- or second-year students were not found to be more depressed, anxious, and stressed than their peers in other years [8, 10, 19–22]. The study also did not reveal that nursing students and other students in the School of Health Sciences Geneva had more risk of poorer mental health than students from psychology, medicine, or pharmaceutical sciences [5–7].

Our analyses indicate, however, that academic satisfaction was stronger than COVID-19 in predicting student psychological health, thus offering additional evidence on the connection between students’ academic satisfaction and mental health. Previous studies were conducted in Korea and in Turkey [19, 39]. In Korea, higher academic satisfaction was associated with less stress, and, in Turkey, satisfied students had lower depression, anxiety, and stress scores than those unsatisfied. What students have identified as deeply satisfying academically include the balance between study and personal life, society’s views of students, feeling able to cope with the workload, the physical condition of the learning environment, the availability of learning resources, feeling able to get financial advice, the variety of assessment techniques, and other students’ views of university life [40]. Academic satisfaction was also found to be influenced by factors such as students’ grades and performance, the program, the quality of teaching, student-to-faculty ratios, and faculty credentials [41–43]. Furthermore, supportive college environments, students’ sense of belonging, civic engagement, and professional confidence allow college students to flourish and positively predict their psychological well-being [44]. Therefore, one can hypothesize how COVID-19 lockdown measures and learning disruption compromised many of the factors contributing to academic satisfaction and mental well-being, such as a sense of belonging, study-life balance, or confidence in one’s performance and future professional outlooks.

In comparing the study conducted at the beginning of the academic year (Franzen et al., 2021) with the present study done at the end of academic year and after the COVID-19 outbreak, several factors may explain the worsening in student mental health in this study compared to Franzen et al. (2021). First, during the academic year-end period and despite the COVID-19 disruptions, all the training institutions involved in this research continued to carry out university tests and exams—a known source of student stress [12–14]. Leniency was, however, applied to exam no-shows and grading to account for the COVID-19 situation.

Second, COVID-19 has been shown to affect negatively the mental health of students worldwide [26–28]. Our study indicates that it mainly worsened stress and anxiety levels, which mirrors another Swiss study, where students reported higher levels of loneliness, stress, and anxiety and decreased social interaction [45]. In a global study covering 62 countries, students expressed anxiety, boredom, frustration, and concerns about their academic and professional careers [46]. In another study from Bangladesh, students reported e-learning burden and fear of missing out [47]. Nursing students in Israel stressed the lack of personal protective equipment as a source of high anxiety [29].

### Implications for policy, practice, and research

Considering COVID-19 upending and the importance of students’ academic satisfaction per se and as a predictor of psychological health, academic institutions must prioritize implementing and evaluating relevant interventions. In the short and medium term, it is critical to implement measures to mitigate the impact of COVID-19 restrictions on students’ learning experience and psychological distress. In the medium and long term, efforts should be made to tackle the factors influencing students’ academic satisfaction directly.

By way of example, establishing blended learning strategies, balancing the unique clinical learning opportunities offered by COVID-19 prevention and control services with proper protective measures and equipment, offering leniency on tests, exams, and deadlines, and ensuring prompt access to quality psychosocial services whenever necessary have been welcome by students [48, 49]. Institutions could also draw from evidence-informed stress management programs, which were developed for medical and nursing students but could benefit those of other health disciplines [50, 51]. Such programs include, for instance, self-hypnosis, meditation, mindfulness-based stress-reduction, feedback on various health habits, educational discussion, changes in the length and type of curriculum, changes in the grading system, or music therapy and muscle relaxation before exams to improve academic performance [50, 51].

### Strengths and limitations

There were several limitations in our study. First, the self-administered survey provided subjective measures. Second, participants’ age, Bachelor’s year distribution, and health discipline distribution differed between the two studies, and the data were not matched, which would have increased power in our analysis by eliminating variation between samples. Given both studies’ anonymous nature, we could not link data at the individual level to offer a longitudinal perspective. However, without the assurance of anonymous data, we could not have carried out the study, given that the ethical committee would not have accepted the study. Third, regarding the lower response rate of the current study compared to our first pre-pandemic survey, we speculate that one of the reasons might have been that students of the current study were forced to follow all their courses online, thereby being much more solicited to use online platforms and more likely to suffer from online fatigue than those of the former study. Fourth, using a control group (students with similar demographic characteristics but not studying health) would have expanded the scope of the current study by allowing for more comparative conclusions. However, the main goal of this research was to look at causes other than psychological distress in a variety of health-related fields (not only nursing and medical students). Fifth, the survey did not focus on the overall stress associated with COVID-19 in students’ life but specifically on COVID-19 as a stressor in the context of their learning experience. Finally, the cross-sectional design could not rule out reverse causality, meaning that lower psychological distress could have resulted in lower COVID-19 stress on learning experience, greater academic satisfaction, or both.

The research had a number of strengths. First, both psychological distress and well-being were examined. Second, it surveyed students in health fields other than medicine, nursing, and psychology. Third, it used a rigorous statistical analysis approach with hierarchical regressions. Finally, using a similar survey among participants from the same academic backgrounds but at two different time points, which were characterized by notable contextual changes, allowed us to draw valuable comparisons.

## Conclusions

Compared to COVID-19 related stress, academic satisfaction had a stronger association with depression, anxiety, stress, and psychological well-being among Bachelor’s students of health disciplines at the end of the academic year. Training institutions should tackle the factors that can catalyze academic satisfaction and ensure that students have a continuous and adequate learning experience despite COVID-19 restrictions. Equally critical is the timely access to relevant psychosocial services to prevent and alleviate mental distress and boost their psychological well-being.
